# Clinical and Molecular Assessment of Patients with Lynch Syndrome and Sarcomas Underpinning the Association with *MSH2* Germline Pathogenic Variants

**DOI:** 10.3390/cancers12071848

**Published:** 2020-07-09

**Authors:** Nathália de Angelis de Carvalho, Bianca Naomi Niitsuma, Vanessa Nascimento Kozak, Felipe D’almeida Costa, Mariana Petaccia de Macedo, Bruna Elisa Catin Kupper, Maria Letícia Gobo Silva, Maria Nirvana Formiga, Sahlua Miguel Volc, Samuel Aguiar Junior, Edenir Inez Palmero, José Cláudio Casali-da-Rocha, Dirce Maria Carraro, Giovana Tardin Torrezan

**Affiliations:** 1 Genomics and Molecular Biology Group, International Research Center/CIPE, A.C.Camargo Cancer Center, São Paulo 01508-010, Brazil; nathalia.carvalho-aluno@accamargo.org.br (N.d.A.d.C.); bianca.niitsuma@accamargo.org.br (B.N.N.); dirce.carraro@accamargo.org.br (D.M.C.); 2 Oncogenetics Service, Hospital Erasto Gaertner, Curitiba 81520-060, Brazil; vanessakozak@hotmail.com (V.N.K.); casali.rocha@accamargo.org.br (J.C.C.-d.-R.); 3 Postgraduate Program in Genetics, Federal University of Parana, Curitiba 81530-000, Brazil; 4 Anatomic Pathology Department, A.C.Camargo Cancer Center, São Paulo 01509-900, Brazil; felipe.costa@accamargo.org.br (F.D.C.); maripetaccia@gmail.com (M.P.d.M.); 5 Colorectal Cancer Department, A.C.Camargo Cancer Center, São Paulo 01509-900, Brazil; bruna.catin@accamargo.org.br (B.E.C.K.); samuel.aguiar.jr@gmail.com (S.A.J.); 6 Radiotherapy Department, A.C.Camargo Cancer Center, São Paulo 01509-900, Brazil; mlgobo@yahoo.com.br; 7 Oncogenetics Department, A.C.Camargo Cancer Center, São Paulo 01509-900, Brazil; nirvana.formiga@accamargo.org.br; 8 Faculdades Pequeno Principe, Curitiba 80230-020, Brazil; sahlua@hotmail.com (S.M.V.); edenirip@yahoo.com.br (E.I.P.); 9 Oncogenetics Department, Barretos Cancer Hospital, Barretos 14784-400, Brazil; 10 Molecular Oncology Research Center, Barretos Cancer Hospital, Barretos 14784-384, Brazil; 11 Instituto de Pesquisa Pelé Pequeno Principe, Curitiba 80250-060, Brazil; 12 National Institute of Science and Technology in Oncogenomics (INCITO), São Paulo 01508-010, Brazil

**Keywords:** Lynch syndrome, sarcoma, *MSH2* germline variant, hereditary cancer, mismatch repair deficiency

## Abstract

Lynch syndrome (LS) is a hereditary cancer-predisposing syndrome associated most frequently with epithelial tumors, particularly colorectal (CRC) and endometrial carcinomas (EC). The aim of this study was to investigate the relationship between sarcomas and LS by performing clinical and molecular characterization of patients presenting co-occurrence of sarcomas and tumors from the LS spectrum. We identified 27 patients diagnosed with CRC, EC, and other LS-associated tumors who had sarcomas in the same individuals or families. Germline genetic testing, mismatch repair (MMR) protein immunohistochemistry, microsatellite instability (MSI), and other molecular analyses were performed. Five LS patients presenting personal or family history of sarcomas were identified (3 *MSH2* carriers and 2 *MLH1*), with 2 having Muir–Torre phenotypes. For two *MSH2* carriers we confirmed the etiology of the sarcomas (one liposarcoma and two osteosarcomas) as LS-related, since the tumors were MSH2/MSH6-deficient, MSI-high, or presented a truncated MSH2 transcript. Additionally, we reviewed 43 previous reports of sarcomas in patients with LS, which revealed a high frequency (58%) of *MSH2* alterations. In summary, sarcomas represent a rare clinical manifestation in patients with LS, especially in *MSH2* carriers, and the analysis of tumor biological characteristics can be useful for definition of tumor etiology and novel therapeutic options.

## 1. Introduction

Lynch syndrome (LS) is a hereditary cancer predisposition syndrome caused by germline mutations in the DNA mismatch repair (MMR) genes *MLH1*, *MSH2*, *MSH6*, and *PMS2* [1], as well as rare deletions in *EPCAM* that disrupt MSH2 expression [2]. The condition is associated mainly with the increased risk of colorectal and endometrial tumor development [3,4], but it is also related to predisposition to several extracolonic malignancies, such as gastric, urothelial, small bowel, ovarian, brain, and hepatobiliary tumors [5]. Within the spectrum of extracolonic tumors related to LS, sebaceous tumors and keratoacanthomas occur in Muir–Torre syndrome (MTS), a LS variant comprising 1–3% of LS cases [6].

Sarcomas are not considered part of the common LS tumor spectrum; however, considering the clinical and molecular aspects of these tumors, a few studies and case reports on patients with LS have described a relationship to them [7,8,9]. Additionally, in a recent study involving 1162 patients with sarcoma who were unselected with regard to family history of cancer, 20% of patients had pathogenic (P) or likely pathogenic (LP) variants in 72 genes associated with the increased risk of cancer, including 11 patients with P or LP variants in MMR genes [10].

Histopathological and molecular diagnostic tests are widely used in LS screening; they include immunohistochemical analysis (IHC) of MMR proteins and microsatellite instability (MSI) testing [4]. The IHC findings of tumors from the LS spectrum, such as colorectal cancer (CRC) and endometrial cancer (EC), usually include the loss of expression of one or two MMR proteins and instability in two or more microsatellite markers [3]. Hence, the evaluation of these specific molecular changes observed in LS-related tumors may be useful for investigation of the etiologies of unexpected tumors in patients with LS [11,12].

Thus, the aims of this study were to evaluate the occurrence of sarcomas in a cohort of patients with tumors on the LS spectrum and to perform clinical and molecular characterization of these patients, identifying molecular features that contribute to elucidate the etiology of these sarcomas. Additionally, we reviewed articles and case reports to elucidate the possible relationship between sarcomas and LS.

## 2. Results

### 2.1. Clinical and Molecular Features of the Patients

We assessed the clinical reports of more than 6600 cancer patients (including 178 LS patients) to search for the co-occurrence of sarcoma and LS-related tumors. We identified 24 patients with sarcomas and LS-spectrum tumors, and 3 cases of these entities in distinct individuals of the same family (Table 1). Among the 24 patients diagnosed with sarcoma and a LS tumor in the same patient, the most frequent LS tumors were CRC (79.2%), EC (16.7%), sebaceoma (8.3%), and renal pelvis and ureter cancer (4.2%). Three patients with genetically diagnosed LS harbored the sarcoma and CRC in different members of the family—one patient had a CRC and a relative diagnosed with sarcoma and two patients had personal history of sarcomas and familial history of CRC tumors. Among the 27 selected patients, 16 reported family history of cancer (7 with LS-related tumors, predominantly CRC and gastric cancer); 4 patients denied any family history of cancer, and in 7 patients information about their family medical histories was not reported (Table 1).

From 24 patients with LS-related tumor and sarcoma in the same individual, 22 were informative for the temporal analysis of the occurrences of those tumors. Based on the age of the diagnoses of sarcoma and LS-related tumors, 8 were considered synchronic (developed in less than year). Among those whose diagnoses intervals were >1 year, in 3 of them the sarcoma was the first tumor diagnosed; in 11 patients the sarcoma occurred after the LS-related tumor. Two patients presented sarcomas that could be defined as radiation-induced tumors—one patient (SLS13) had a previous whole body radiation exposure (Hiroshima atomic bomb survival), while the second patient (SLS3) developed a sarcoma 9 years after radiation therapy for a rectum adenocarcinoma, in the field of the radiation therapy (Table 1).

Out of twelve colorectal tissue samples subjected to IHC analysis, eight were MMR-intact and four showed MMR loss (one in MSH2/MSH6 and three in MLH1/PMS2). Additionally, one patient had a previous result of MSH2/MSH6-deficient sebaceoma. Five patients had been confirmed through previous genetic testing to be carriers of germline pathogenic (P) or likely pathogenic (LP) variants in MMR genes (three in *MSH2* and two in *MLH1*), and two of these patients had the MTS phenotype. Two patients fulfilled the Amsterdam I clinical criteria (Table 1).

In six cases, we were able to perform MMR-IHC or MSI analysis of the sarcoma tissue. Two LS patients (case SLS1, described in detail in the next section, and case SLS26) presented sarcomas with loss of MSH2/MSH6 and with high microsatellite instability (MSI-H). Case SLS26 was a carrier of the *MSH2* pathogenic variant and presented two primary sarcomas—a liposarcoma at 18 years of age and an osteosarcoma at 20 years of age. The liposarcoma presented loss of MSH2/MSH6 on IHC and both sarcomas were MSI-high (Idylla™ MSI assay).

Two patients (cases SLS6 and SLS23) presented loss of MLH1/PMS2 expression only in the CRC, while their sarcomas were MMR-intact (SLS6) or microsatellite stable (MSS) (SLS23). We performed a germline multigene panel testing in patient SLS6 and we identified a variant of unknown significance (VUS) in the *TP53* gene (p.Asp49His). We also confirmed that the MLH1/PMS2 loss in this patient’s CRC was due to somatic *MLH1* promoter methylation (Figure S1). Although additional germline genetic testing was not done in patient SLS23, the CRC harbored a somatic mutation in *BRAF* (p.Val600Glu), which is usually associated with somatic *MLH1* promoter methylation and the sporadic CRC pathway [13]. 

We performed germline multigene testing containing 94 or 113 genes in 14 patients without previous results. A few incidental variants not related to LS phenotypes were identified in these patients. We detected a total of 26 rare variants, including two pathogenic (P) and likely pathogenic (LP) loss of function variants in *RAD51C* and *FANCE* in case SLS19 (Table 1) and 24 VUS) in 11 patients (Table S1). The VUS were detected in 20 known cancer predisposing genes, including *BRCA2, CHEK2, RECQL4, SDHB, SDHD*, and *TP53*, which have already been linked to an increased risk of sarcoma.

### 2.2. Molecular Characterization of the MSH2 c.1661+1G>A Carrier

**Figure 1:**
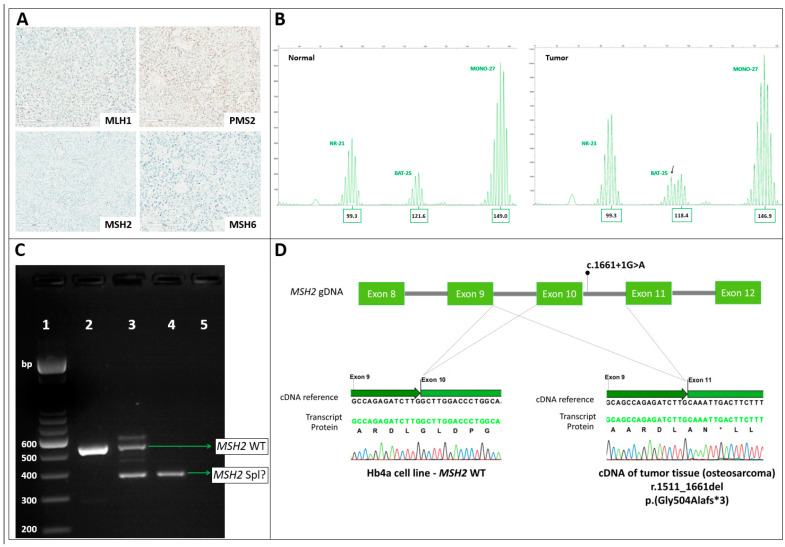
Molecular characterization of the patient SLS1. (**A**) IHC results for the four MMR proteins in the osteosarcoma, showing the nuclear retained positive expression of MLH1 and PMS2 and the loss of expression of MSH2 and MSH6. (**B**) Results of MSI analysis by capillary electrophoresis. The base pair sizes of the major alleles are shown below each marker. For the BAT-25 marker, a new allele (arrow) is evident in the tumor tissue. NR-21 is stable and MONO-27 shows subtle changes of 2 bp in the tumor tissue, indicated by the changes in the heights of the main peaks and the stutter patterns. BAT-26 and NR-24 also exhibited subtle instability (data not shown). (**C**) RT-PCR products amplified from exons 8–12 of the MSH2 transcript, demonstrated on 2% agarose gel; (1) ladder (100 bp); (2) positive control (HB4a cell line) showing the wildtype MSH2 transcript (532 bp); (3) adjacent normal tissue from SLS1 showing the wild-type transcript (532 bp) and an aberrant transcript (400 bp); (4) osteosarcoma tissue from SLS1 showing only the aberrant transcript (400 bp); (5) negative control. (**D**) Sanger sequencing of RT-PCR products from the control cell line and the patient osteosarcoma’s tissue revealed the skipping of MSH2 exon 10 in the aberrant transcript identified in SLS1 tissues.

One of the identified patients (SLS1) had a probably pathogenic germline variant in *MSH2* (c.1661+1G>A). We investigated the relation of the patient’s sarcoma diagnosis with the germline *MSH2* variant through IHC and molecular analysis of the tumor sample. The results of IHC showed loss of MSH2/MSH6 protein expression in the osteosarcoma (Figure 1A) and the MSI analysis detected high instability (MSI-H), with four out of five microsatellites being unstable and with subtle size changes (Figure 1B). As is frequently the case, the interpretation of allelic profiles from this sample with subtle (1- and 2-bp) changes was difficult due to the presence of stutters (PCR artifacts produced by repeat slippage); only one marker presented clear >2-bp instability. For this reason, we confirmed the MSI-H pattern of this case using the Idylla™ MSI assay, which revealed that five out of seven markers were unstable.

We also sought to confirm the pathogenicity of the LP splice site variant in *MSH2* identified in this patient, as no confirmatory aberrant transcript has been described in the literature. We, thus, performed transcript analysis of *MSH2* of the osteosarcoma and adjacent normal tissues. Sanger sequencing of RT-PCR products revealed an aberrant transcript involving the complete skipping of exon 10 in the tumor and normal tissues (Figure 1C,D), which resulted in a premature stop codon at codon 507 (p.Gly504Alafs*3). Our results allowed the reclassification of this variant from LP to P, according to American College of Medical Genetics (ACMG) guidelines. Additionally, Sanger sequencing of the tumor and blood DNA revealed significant reduction of the normal allele peak in the tumor, indicating that the second *MSH2* inactivation event probably occurred due to deletion of the normal allele, causing loss of heterozygosity (LOH; Figure S2).

### 2.3. Reports of Sarcomas in Patients with LS

The literature review led to the identification of 22 studies of interest [6,7,8,9,11,14,15,16,17,18,19,20,21,22,23,24,25,26,27,28,29,30,31], reporting on 43 cases of sarcoma associated with LS (Table 2 and Table 3). Most tumors in these cases showed negative expression of MSH2 or MSH2/MSH6 (*n* = 19) on IHC analysis. Isolated MSH6 loss was observed in 3 tumors, and MLH1 or MLH1/PMS2 loss were observed in 6 tumors. Germline mutations in the *MSH2* gene (25 patients) were described more frequently than those in other MMR genes (*MLH1* in 12 patients, *MSH6* in 5 patients and PMS2 in 1 patient). MSI status was described for 20 of 43 cases, with 12 cases being MSI-H, 2 cases being MSI-I (indeterminate), and 6 being MSS (Table 2 and Table 3).

Remarkably, the concordance of germline pathogenic variants and loss of protein expression or presence of MSI was higher for the *MSH2* gene, since 87% (21/24) of sarcomas from *MSH2* carriers harbored one of these hallmarks, in contrast to 75% for *MLH1* (6/8) and *MSH6* (3/4) and 0% for *PSM2* (0/1) (Table 3).

## 3. Discussion

Sarcomas comprise an extensive and heterogeneous group of rare mesenchymal neoplasms, with more than 50 described histopathological subtypes, representing around 1% of all malignancies in adults [7,32]. These tumors have been described in families affected by LS and germline variants in MMR genes have also been identified in unselected sarcoma cases, however no definitive association or defined risk related to this syndrome has been established. In this study, we searched our institutional databases to identify the co-occurrence of sarcoma in patients or families with tumors from the LS spectrum, and identified five such cases involving LS. For two patients, we confirmed that the etiology of the sarcomas was related to LS by performing multiple molecular analyses (MSI, IHC, transcript sequencing, and LOH analyses). In one case, we also showed that the *MSH2* splice-site variant generated a truncated transcript, unequivocally confirming its pathogenicity.

A few previous reports have also described the development of sarcomas in patients with LS associated with loss of MMR protein expression in the tumors, thereby relating the etiology to germline mutations in MMR genes [8,9,11,17,30]. Clinical and pathological or molecular data from reports identified in our literature review suggest that the development of sarcoma is more often associated with *MSH2* pathogenic variants than with other LS genes, as 25 out of 43 (58.1%) cases subjected to genetic testing harbored mutations in this gene. This frequency is higher than observed in most populations, in which *MSH2* is typically the second most frequently mutated gene (seen in 40–50% of cases) [33,34]. In our cohort, three of the five patients with LS harbored *MSH2* pathogenic variants.

In addition, two of the *MSH2* carriers in our study had MTS, a rare variant of LS presenting with sebaceous gland neoplasims. Reports from the literature describe three other patients diagnosed with MTS and sarcomas. Lee et al. [8] described a patient with MTS and high-grade undifferentiated pleomorphic sarcoma presenting MSH2 loss of expression and MSI-H. Yozu et al. [5] described a patient with MTS and pleomorphic liposarcoma presenting loss of MSH2/MSH6 expression and a confirmed *MSH2* germline pathogenic variant. Latham et al. [24] described a patient with an MSI-H soft-tissue sarcoma and sebaceous adenoma who was a carrier of the *MSH2* pathogenic variant. The *MSH2* gene is known to be more strongly associated with MTS and other extracolonic tumors than are other LS-related genes [35,36], and it also seems to be more associated with an increased risk of sarcoma. 

In this sense, a recent publication based on The Cancer Genome Atlas data for cancer-predisposing variants described the detection of two *MSH2* carriers among 255 unselected patients with sarcoma and the classification of *MSH2* as potentially associated with sarcoma risk according to variant burden analysis (odds ratio, 9.9; *p* = 0.02; false discovery rate, 0.09) [37]. Moreover, studies performed with the Prospective Lynch Syndrome Database also indicate an increased lifetime incidence of sarcomas, linking these tumors to LS tumor spectrum [34,38]. Although these articles did not describe specific risks of sarcomas in LS patients, nor the affected gene in these cases, the most recent study of this group including 6350 LS patients with extended follow-up (51,646 years) have described 12 cases of osteosarcoma and 4 cases of soft-tissue sarcoma. Compared to regular population incidences (0.34/100,000 for osteosarcomas [39] and 3.4/100,000 for soft-tissue sarcomas [40]), this indicates that osteosarcoma and soft-tissue sarcoma incidences are >50 timers and >1.8 times higher, respectively, in LS patients. Reinforcing this increased risk of osteosarcomas, recently Mirabello et al. [41] investigated germline causes of 1244 patients with osteosarcoma and identified more germline *MSH2* pathogenic variants in cases than in the control cohorts (*p* < 0.05).

Other cancer-predisposing syndromes may be related to the co-occurrence of sarcoma and common LS-related tumors. Among 14 patients in our cohort who were evaluated with expanded gene panels, only one patient carried P/LP variants in non–LS-related genes. This patient was diagnosed with a gastrointestinal stromal tumor (GIST) and rectal adenocarcinoma and carried germline P/LP variants in *FANCE* and *RAD51C*. Neither of these genes is known to be associated with an increased risk of sarcoma or CRC development, and the relevance of these variants in this case remains unclear.

Besides cancer-predisposing syndromes, other genetic and environmental risk factors may explain the co-occurrence of sarcomas and LS-related tumors in the same individuals. Previous exposure to radiation has long been known to play a causative role in the pathogenesis of sarcomas, with radiation-induced sarcomas (RISs) constituting about 2–5% of all sarcomas [42,43,44,45]. RISs have various histological types and usually develop after latency periods of 2–10 years, although the minimal latency period required for the designation of a tumor as radio-induced remains under debate [45,46]. In our study, 2 of 25 patients had tumors that could be classified as RISs. One case was a high-grade undifferentiated pleomorphic sarcoma in the radiation field more thar 9 years after the after the treatment of rectal carcinoma. The other probable RIS was a low-grade retroperitoneal leiomyosarcoma occurring in a Japanese survivor of the Hiroshima atomic bombing. Finally, it is also possible that in these two patients, a combination of genetic predisposition and radiation exposure may have contributed to the development of multiple primary tumors; however, this hypothesis could not be evaluated because these patients did not undergo genetic testing.

Sarcoma development has been linked to germline and somatic mutations in MMR genes, not only in patients with clinical phenotype of LS, but also in unselected cohorts of patients. In this sense, MSI and MMR IHC analyses are valuable tools for the identification of tumors originating from MMR pathway defects (both somatic and constitutive), and may aid the detection of undiagnosed LS in patients with unexpected phenotypes. Early studies addressing this issue were performed with small cohorts of sporadic sarcomas and heterogeneous histopathological subtypes, and involved various detection techniques; they produced inconsistent results, but the majority showed that most sarcomas do not present MMR deficiency [8,47,48].

Recently, the advent of immune checkpoint inhibitor therapy and its striking efficiency for MMR-deficient tumors, regardless of tumor site [49,50], prompted demand for the detection of MMR deficiency in various tumor types. In this scenario, Latham et al. [24] used next-generation sequencing (NGS) data to evaluate MSI in 15,045 patients diagnosed with more than 50 cancer types. The overall rate of MSI-H or indeterminate MSI in soft-tissue sarcomas was 5.7% (45/785 patients), and 2 of these 45 patients had LS due to *MSH2* pathogenic variants. In another recent NGS study, MMR deficiency was identified in 2.3% (7/304) of sarcomas (4 unclassified sarcomas, 1 pleomorphic rhabdomyosarcoma, 1 epithelioid leiomyosarcoma, and 1 malignant perivascular epithelioid cell neoplasm). Somatic mutation analysis identified MMR mutations in all seven patients (four in *MSH2* or *EPCAM*, two in *PMS2*, and one in *MSH6*); germline sequencing was performed for three patients and confirmed LS in one patient (a carrier of a *MSH2* germline pathogenic variant) [23]. Lastly, the clinical importance of recognizing LS carriers among patients with sarcoma was highlighted recently by the case reported by Tlemsani et al. [22]. The authors described a 19-year-old patient who presented with a metastatic chemoresistant pleomorphic rhabdomyosarcoma. An incidental finding of a germline pathogenic variant in *MLH1*, identified through whole-exome sequencing, led to the administration of anti-PD-1 antibody treatment (nivolumab), which resulted in a rapid major response.

We acknowledge that our study has some limitations. First, since we did not investigate the contribution of germline variants in LS genes in a unbiased sarcoma cohort and we had a small number of the LS patients, this study does not have the required power or design to establish the risk of sarcoma development in LS patients. Additionally, the family history described for our patients was based on patients’ verbal reports. The evidence presented in our study highlight that molecular features can contribute to elucidating the etiology of sarcomas in LS cases and reinforce the already hypothesized causal link between sarcomas and MMR defects.

## 4. Materials and Methods 

### 4.1. Patients

First, we evaluated the personal and family history of cancer of Lynch syndrome patients from three cancer hospitals, namely A.C. Camargo Cancer Center (ACCCC), Barretos Cancer Hospital (BCH), and Erasto Gaertner Hospital (EGH), in order to identify LS patients/families with sarcoma diagnosis. In total, 178 LS patients (confirmed to be carriers of germline pathogenic variants) were evaluated (101 from ACCCC, 67 from BCH and 10 from EGH).

Second, at ACCCC we used RECRUIT, an internal database to search for cancer patients treated at ACCCC that were diagnosed with sarcomas and tumors of the LS spectrum (CRC, EC, urinary tract carcinoma, and sebaceous carcinoma). The patients were identified by filtering of electronic medical records in the database using the following terms: “sarcoma” AND ("colon adenoma” OR “colon adenocarcinoma” OR “colorectal cancer” OR “endometrial cancer” OR “renal pelvis” OR “urothelial” OR “sebaceous”). This database is a search tool that identifies the defined key words in electronic medical records from ACCCC patients. In total 4816 patients’ records were detected in RECRUIT and manually inspected to identify patients diagnosed with tumors of the LS spectrum and sarcomas (in the same individual or in family members). Additionally, we evaluated clinical records of patients with loss of MMR protein expression in CRC or sebaceous tumors from curated databases of the Department of Pelvic Surgery (containing 1654 patients) and the database from the Anatomic Pathology Department (containing 14 patients) to identify any co-occurrence with sarcomas. 

The following clinical data were collected for each included patient: age of onset, other primary tumors, tumor histology, family history of cancer, and previous results of MMR IHC analysis and germline genetic testing. Patients who had not undergone previous genetic testing were evaluated to detect germline variants in cancer-predisposing genes. Genomic DNA from these patients was obtained from the ACCCC biobank or prospectively through saliva collection. For one patient diagnosed with osteosarcoma who had a LP germline variant in *MSH2* intron 10 (c.1661+1G>A), a complete molecular analysis of the tumor was performed, including MSI analysis and transcript analysis using Sanger sequencing. Patients lacking information regarding MMR protein expression or MSI status and available tumor material were evaluated for these.

Prospectively included patients provided written informed consent. This study was performed in accordance with the Helsinki Declaration and was approved by each corresponding institutional research board (IRB): ACC IRB approval 90018418.2.0000.5432, EGH IRB approval 65971917.0.0000.0098 and BCH IRB approval 23350513.0.0000.5437.

### 4.2. Immunohistochemical Analysis of Mismatch Repair Proteins

The expression of MMR proteins (MLH1, MSH2, MSH6, and PMS2) in formalin-fixed, paraffin-embedded (FFPE) tissues from CRCs, ECs, and sarcomas was evaluated using standardized IHC protocols applied in the clinical routine at the Anatomic Pathology Department of ACCCC. The following antibodies were used: MLH1 (M1; Ventana), MSH2 (G219-1129; Cell Marque), MSH6 (44; Ventana), and PMS2 (EPR3947; Cell Marque). Loss of nuclear expression was considered when tumor cells showed complete absence of expression for one or more markers, with adequate positive internal control in stromal or inflammatory cells.

### 4.3. Microsatellite Instability

The MSI analysis was performed using the MSI analysis system kit (version 1.2; Promega, Madison, WI, USA), following the manufacturer’s standard protocol. Briefly, five mononucleotide microsatellite markers (BAT-25, BAT-26, NR-21, NR-24, and MONO-27) were amplified by polymerase chain reaction (PCR) using fluorescent primers, and the amplified products were evaluated in an automated DNA-sequencing genetic analyzer (model 3500; Thermo Fisher Scientific, Waltham, MA, USA). The electrophoretic pattern of the tumor DNA was compared with the DNA pattern of normal tissue. Classification was performed according to the frequency of marker instability, with microsatellite stability (MSS) defined as stability of all markers, a high degree of microsatellite instability (MSI-H) defined as instability of two or more of markers, and a low degree of microsatellite instability (MSI-L) defined as instability of a single marker, following the suggestion of the National Cancer Institute [51].

Alternatively, the MSI status was evaluated using the Idylla™ MSI assay (Biocartis, Mechelen, Belgium), performed on the Idylla™ system (Biocartis). This automated method, based on high-resolution melting PCR, detects MSI by analyzing a panel of seven monomorphic microsatellites in FFPE tumor tissue samples (no normal tissue is required). Classification is based on the number of markers identified as unstable, with MSS defined as instability of no or one marker and MSI-H defined as instability of two or more markers.

### 4.4. Transcript Analysis 

The transcript analysis was performed using *MSH2* gene transcripts of RNA extracted from tissue samples from frozen tumors and normal adjacent tissue of the proband (carrier of *MSH2* c.1661+1G>A). RNA from the Hb4a cell line was used as the normal control. The cDNAs were synthesized by reverse transcriptase (RT) reaction from l µg of total RNA using oligoDT and the SuperScript III enzyme (Invitrogen, Carlsbad, CA, USA). The cDNA was used for RT-PCR, with primers in exons 8 and 12 used to generate fragments that potentially contained splicing variants. The RT-PCR products were visualized on 2% agarose gel stained with SYBR Safe (Invitrogen) under ultraviolet light with a transilluminator (Alpha Innotec, BY, DE). The amplified products were sequenced using the BigDye^®^ Terminator kit (version 3.1; Applied Biosystems, Foster City, CA, USA), and automated DNA sequencing was performed with an ABI Prism 3130 XL system (Applied Biosystems), according to the manufacturer’s instructions. The resulting sequences were analyzed using the CLC Main Workbench software (CLC Bio, version 6, Aarhus, DK).

### 4.5. MLH1 Promoter Methylation Analysis

Methylation analysis of the *MLH1* promoter was performed by next-generation sequencing (NGS) of bisulfite-converted DNA. Briefly, DNA extracted from fresh-frozen tumor was treated with the EZ DNA Methylation Gold kit (Zymo Research, Orange County, CA, USA) and subjected to PCR using primers for the promoter region of MLH1 (extending from −209 to −181 of the MLH1 transcription initiation site). Primer sequences and PCR conditions are available under request. The PCR products were evaluated on agarose gel and used to construct amplicon libraries with the Ion Plus Fragment Library kit (Thermo Fisher Scientific). Emulsion PCR and sequencing were performed using the Ion PI™ Hi-Q™ OT2 200 kit, Ion PI™ Hi-Q™ Sequencing 200 kit, and Ion PI™ Chip kit (version 3; Thermo Fisher Scientific), according to the manufacturer’s recommendations. The sequences generated by the ion proton sequencer were mapped in the MLH1 converted reference sequence using the CLC Genomics Workbench software (CLC Bio). Cytosine positions not adjacent to guanines were used to evaluate the bisulfite conversion efficiency, and five specific CpG regions were used to calculate the average methylation level of the MLH1 promoter.

### 4.6. Multigene Panel Germline Sequencing

Genomic DNA was extracted from peripheral blood or saliva samples at the ACCCC biobank. The DNA was submitted to hybridization-based capture using the 94-gene TruSight cancer panel (Illumina, San Diego, CA, USA) or a customized 113-gene panel (xGen Gene Capture Pools; IDT Technologies, Coralville, IA, USA; Table S1), according to the manufacturer’s recommendations. Sequencing was performed with a NextSeq 500 system (Illumina). Variant calling was performed using the Enrichment plugin in BaseSpace (Illumina), and VCF files were annotated and filtered using VarSeq software (Golden Helix, version 2.0, Bozeman, MT, USA). Identified variants were classified following the American College of Medical Genetics and Genomics (ACMG) and the Association of Molecular Pathology (AMP) guidelines recommendations [52].

### 4.7. Literature Review

Publications from Medline database were searched using the PubMed resource. The searches were performed using the following terms and keywords: ((sarcoma) AND (Lynch)) OR ((sarcoma) AND (HNPCC)); and ((sarcoma) AND (PMS2)) OR ((sarcoma) AND (MLH1)) OR ((sarcoma) AND (MSH6)) OR ((sarcoma) AND (MSH2)). The 295 resulting articles were filtered manually to assess whether they reported cases of patients with sarcomas and confirmed LS diagnosis (by either germline genetic testing results or complete information of family history, IHC, and MSI analysis). Cases with a clinical suspicion of LS but without an extensive molecular analysis were not considered in this literature review. LS cases with sarcomas but without information regarding the affected gene were not included. Clinical data describing the age of onset, tumor histology, MMR IHC analysis, MSI status, and germline genetic testing or affected genes were collected for each reported case. 

### 4.8. Data Availability

The data that support the findings of this study are available from the corresponding author upon reasonable request.

## 5. Conclusions

Our results and data from cases described in the literature indicate that sarcomas are rare clinical manifestations of LS, especially in MSH2 pathogenic carriers. Although the risk of sarcoma is clearly much lower than that for other tumors from the LS spectrum and still needs to be defined, clinicians need to recognize this association so that they can offer proper genetic counseling and risk awareness for both families affected by clinically defined LS and for patients with unexpected genetic LS diagnosis identified through tumor molecular profiling. Finally, the identification of sarcoma in a family affected by LS should not be regarded as a sporadic finding, but rather as a possibly MMR-deficient tumor, which can be confirmed by molecular analysis and has clinical implications, especially considering the recent available immunotherapies.

## Figures and Tables

**Table 1:** Clinical and molecular data from patients diagnosed with sarcoma and tumors from the LS spectrum.

Patient	Sex	Tumor Histology (Sarcoma), Age at Diagnosis	Tumor Histology (CRC), Age at Diagnosis	MMR IHC (CRC)	MMR IHC; MSI Status (Sarcoma)	Other Tumors	Genetic Testing (P/LP Variants)	Family History (Site, Age at Diagnosis)
SLS1	F	Osteosarcoma, 40	No CRC	-	MSH2/MSH6 loss; MSI-H	**Sebaceoma** (MSH2/MSH6 loss)	Lynch syndrome/MTS (*MSH2* c.1661+1G>A-LP)	Sister (CRC, 45), mother (CRC, 50), niece (CRC, 41) ^a^
SLS2	F	Primary high-grade undifferentiated pleomorphic sarcoma of the lung, 67	Focal carcinoma in situ in rectal villous adenoma, 68	NA	NA	Lung adenocarcinoma, 68	Not detected	Father (NS, 84), brother (liver, 55), maternal cousin (breast, 60)
SLS3	M	High-grade undifferentiated pleomorphic sarcoma in colon ^b^, 50	Moderately differentiated adenocarcinoma of the rectum, 41	Intact	NA	-	NA	Patient denies any family history of cancer
SLS4	F	High-grade sarcoma, 75	Moderately differentiated, adenocarcinoma, ulcerated and infiltrating to serous, 72	Intact	NA	-	Not detected	Patient denies any family history of cancer
SLS5	F	High-grade leiomyosarcoma, 57	Well-differentiated tubular adenocarcinoma, 55	Intact	NA	-	Not detected	Patient denies any family history of cancer
SLS6	M	Grade 2 pleomorphic sarcoma, 82	Upper rectum Adenocarcinoma, 68 Signet ring cells adenocarcinoma, 82	MLH1/PMS2 loss	Intact; NA	-	Not detected	Father (GC, 70), first-grade nephew (CRC, 75), brother (GC, NA)
SLS7	M	Grade 2 myxofibrosarcoma, 64	Tubulovillous Adenoma with Intramucosal Adenocarcinoma, 66	Intact	NA	-	NA	NA
SLS8	F	No sarcoma	Invasive moderately differentiated adenocarcinoma, 36	MLH1/PMS2 loss	NA	Ovarian germ cell tumor, 29	Lynch syndrome (*MLH1* c.2004delA; p.Glu669Lysfs*114-P)	Mother (osteosarcoma, 37), paternal grandfather (oropharynx, NA), paternal uncle (prostate, 62), paternal aunt (lung, 69)
SLS9	F	Low-grade liposarcoma, 65	Rectal adenocarcinoma, 70	Intact	NA	-	Not detected	Maternal grandmother (CRC, NA), maternal aunt (bowel, NA), maternal uncle (CRC, NA), maternal aunt (GC, NA)
SLS10	F	Low-grade endometrial stromal sarcoma, 42	Well-differentiated tubular adenocarcinoma, 39	Intact	NA	Thyroid tumor, 46	Not detected	Sister (thyroid, 51), father (bladder, 63), maternal grandfather (renal, 55), maternal uncle (esophagus, 53)
SLS11	M	Soft-tissue sarcoma, 51	Well-differentiated tubular adenocarcinoma, 53	MSH2/MSH6 loss	NA	Sebaceoma	Lynch syndrome/MTS (*MSH2* c.1444A>T; p.Arg482Ter-P)	Brother (CRC, 45), mother (CRC, 50), maternal grandmother (CRC, NA), maternal grandfather (stomach, NA, prostate, NA), maternal uncle (CRC, 40), maternal uncle (CRC, 45) ^a^
SLS12	M	Well-differentiated retroperitoneal liposarcoma, 68	Adenocarcinoma in situ, 67	NA	NA	Liver carcinoma, 68	NA	Mother (liver, NA), son (liver, NA), son (lung, NA)
SLS13	F	Low-grade retroperitoneal leiomyosarcoma ^b^, 72	Sigmoid adenocarcinoma, 61	NA	NA	-	NA	Patient was not able to answer
SLS14	F	High-grade vaginal fusocellular sarcoma, 58	No CRC	-	NA	Endometrial adenocarcinoma (MMR intact), 57	Not detected	Sister (neck, uterus and ovary, 40)
SLS15	F	Dedifferentiated retroperitoneal liposarcoma, 73	No CRC	-	NA	Endometrial adenocarcinoma (MMR intact), 57; Bladder sarcomatoid carcinoma, 81	Not detected	Father (Head and Neck, 75)
SLS16	F	High-grade pleomorphic sarcoma, 80	No CRC	-	NA	Endometrial adenocarcinoma, 70; Breast carcinoma, 87	Not detected	Two daughters (breast and thyroid, NS)
SLS17	F	Pleomorphic sarcoma with giant cells, 71	No CRC	-	NA	Endometrioid adenocarcinoma, 71	NA	Father (CRC, 74), daughter (parotid, 41)
SLS18	F	High-grade dedifferentiated liposarcoma with myxoid, fusocellular and pleomorphic areas, 77	Well-differentiated colon adenocarcinoma, 76	NA	NA	-	Not detected	NA
SLS19	M	Gastrointestinal stromal sarcoma (GIST), 59	Moderately differentiated rectal adenocarcinoma, 60	Intact	NA	-	(*FANCE* c.929dupC; p.Val311Serfs*2-LP); (*RAD51C* c.709C>T; p.Arg237Ter-P)	Mother (uterus, 56)
SLS20	F	Biphasic synovial sarcoma, 76	Moderately differentiated sigmoid carcinoma, 79	Intact	NA	-	Not detected	NA
SLS21	M	Gastrointestinal stromal sarcoma (GIST), 65	Transverse colon adenocarcinoma, 65	NA	NA	Pyriform sinus carcinoma, 72	Not detected	Brother (prostate, NS)
SLS22	F	Poorly differentiated carcinosarcoma, 58	Poorly differentiated sigmoid adenocarcinoma, 59	NA	Intact; NA	Ovarian carcinoma, 54; Vaginal adenocarcinoma, 58	NA	NA
SLS23	F	Low grade superficial leiomyosarcoma, 69	Moderately differentiated colon adenocarcinoma, 70	MLH1/PMS2 loss	NA; MSS	-	NA	Mother (uterus, NS), sister (GC, NS), sister (breast, 40)
SLS24	F	Gastrointestinal stromal sarcoma (GIST), 73	Moderately differentiated rectosigmoid adenocarcinoma, 71; Moderately differentiated cecum adenocarcinoma, 71	NA	NA	-	NA	NA
SLS25	M	High-grade leiomyosarcoma, 62	Tubular adenoma with low grade dysplasia, 59	NA	NA	Papillary urothelial carcinoma of the renal pelvis, 63; Urothelial carcinoma in situ of distal ureter, 63	NA	NA
SLS26	F	Liposarcoma, 18 Osteosarcoma, 20	No CRC	-	MSH2/MSH6 loss; MSI-H	-	Lynch syndrome (*MSH2* c.2152C>T; p.Gln718Ter-P)	Grandfather (CRC, NS), cousin (CRC, 27)
SLS27	F	Myxoid Liposarcoma, 64	No CRC	-	Intact	-	Lynch syndrome (*MLH1* exon 17 to 19 deletion-P)	Daughter (CRC, 32), father (CRC, NS), grandfather (CRC, NS)

Note: LS, Lynch syndrome; CRC, colorectal cancer; MMR, mismatch repair; IHC, immunohistochemistry; MSI, microsatellite instability; MSS, microsatellite stability; MSI-H, high microsatellite instability; P, pathogenic; LP, likely pathogenic; F, female; NA, not available; M, male; GC, gastric cancer; NS, not specified; (**a**) patients fulfilling Amsterdam I clinical criteria; (**b**) possibly radio-induced sarcomas.

**Table 2:** Clinical and molecular features of sarcoma cases in LS patients from literature review.

Study	Age at Diagnosis, Sex	Tumor Histology ^a^	MMR IHC	MSI Status	Germline Variants ^b^ (MMR Genes)
Sijmons et al. (2000) [27]	45, M	Malignant fibrous histiocytoma	MSH2 loss	MSI-H	*MSH2* p.(Gly429Ter)
den Bakker et al. (2003) [28]	23, F	Rhabdomyosarcoma	MSH2 loss	MSI-H	*MSH2* ^c^
Lynch et al. (2003) [29]	25, M	Osteosarcoma	NA	MSI-H	*MSH2* exon 4 splice site mutation
	NA, F	Malignant fibrous histiocytoma	NA	MSI-H	*MSH2* del exon 3–8
Hirata et al. (2006) [30]	40, M	Liposarcoma	MSH2 loss	NA	*MSH2* c.677delAT; p.(Arg227Glufs*19)
South et al. (2007) [31]	46, F	Carcinosarcoma	MLH1 loss	NA	*MLH1* c.1896G>C; p.(Glu632Asp)
Geary et al. (2008) ^d^ [14]	36, M	Soft-tissue sarcoma NS	MLH1 loss	NA	*MLH1* ^c^
	NA	Sarcoma NS	NA	NA	*MSH2* c.1986delG; p.(Met663Cysfs*22)
Nilbert et al. (2009) [7]	27, F	Sarcoma NS	NA	NA	*MSH2* c.145_148delGACG; p.(Asp49Argfs*14)
	38, M	Liposarcoma	MSH2/MSH6 loss	MSS	*MSH2* c.942+3A>T
	24, F	Sarcoma NS	NA	NA	*MSH2* c.942+3A>T
	44, F	Carcinosarcoma	MSH2/MSH6 loss	MSI-H	*MSH2* c.1165C>T; p.(Arg389Ter)
	32, M	Gliosarcoma	MSH2/MSH6 loss	NA	*MSH2* c.1696_1697delAAinsG; p.(Asn566Valfs*24)
	55, M	Liposarcoma	MSH2/MSH6 loss	NA	*MSH2* c.1-?_366+?del
	32, M	Gliosarcoma	MSH2/MSH6 loss	NA	*MSH2* c.1696_1697delAAinsG; p.(Asn566Valfs*24)
	44, F	Leiomyosarcoma	MSH2/MSH6 loss	MSI-H	*MSH2* ^c^
	28, M	Chondrosarcoma	Intact	NA	*MLH1* c.1204A>T; p.(Lys402Ter)
	45, M	Sarcoma NS	NA	NA	*MLH1* c.1204A>T; p.(Lys402Ter)
	15, F	Osteosarcoma	NA	NA	*MLH1* c.1276C>T; p.(Gln426Ter)
	66, F	Liposarcoma	NA	NA	*MLH1* c.1732+?_c.2268del
	55, F	Carcinosarcoma	MSH6 loss	NA	*MSH6* c.1085delC; p.(Pro362Leufs*9)
	74, M	Leiomyosarcoma	NA	NA	*MSH6* c.3514_3515insA; p.(Arg1172Lysfs*5)
	50, M	Malignant hemangiopericytoma	Intact	NA	*MSH6* c.3850_3851insATTA; p.(Thr1284Asnfs*6)
Yu et al. (2009) [16]	40, M	Leiomyosarcoma	MLH1 loss	MSI-H	*MLH1* c.200G>A; p.(Gly67Glu)
Brieger et al. (2011) [17]	43, M	Malignant fibrous histiocytoma	MSH2 loss	MSI-H	*MSH2* c.2038C>T; p.(Arg680Ter)
	50, M	Malignant fibrous histiocytoma	MSH2 loss	MSI-H	*MSH2* c.942+3A>T
Urso et al. (2012) [8]	43, M	Leiomyosarcoma	MSH2/MSH6 loss	MSS	*MSH2* del exon 1–16
Lee et al. (2013) [9]	58, M ^e^	Undifferentiated pleomorphic sarcoma of the retroperitoneum	MSH2 loss	MSI-H	*MSH2* ^c, f^
Yozu et al. (2013) [10]	74, M ^e^	Pleomorphic liposarcoma	MSH2/MSH6 loss	NA	*MSH2* ^c^
Jie et al. (2014) [11]	71, F	High-grade sarcoma NS	MSH2 loss	NA	*MSH2* p.(Arg621Ter)
Schiavi et al. (2015) [18]	37, F	Leiomyosarcoma	MSH2/MSH6 loss	NA	*MSH2* c.649dupA; p.(Ile217Asnfs*15)
	39, M	Leiomyosarcoma	NA	NA	*MLH1* c.2195_2198dupAACA
Nguyen et al. (2016) [19]	35, M	Myxofibrosarcoma	MLH1/PMS2 loss	MSI-H	*MLH1* c.678-7_686del16
Carnevali et al. (2017) [20]	41, F	Ovary Carcinosarcoma	MSH6 loss	MSI-H	*MSH6* c.931_935delAAAAG p.(Lys311Glufs*4)
Saita et al. (2018) [21]	80, F	Sarcoma NS	Intact	NA	*MLH1* ^c^
Tlemsani et al. (2018) [22]	19, M	Rhabdomyosarcoma	MLH1/PMS2 loss	MSS	*MLH1* c.1863_1864insT; p.(Leu622Serfs*10)
Doyle et al. (2019) [23]	36, M	Pleomorphic rhabdomyosarcoma	MSH2/MSH6 loss	NA	*MSH2* p.(Tyr678Ter)
Latham et al. (2019) [24]	71, NA ^e^	Soft-tissue sarcoma	MSH2/MSH6 loss	MSI-I	*MSH2* c.1216C>T; p.(Arg406Ter)
	32, NA	Soft-tissue sarcoma	MSH2/MSH6 loss	MSI-I	*MSH2* c.229_230delAG; p.(Ser77Cysfs*4)
	NA	Soft-tissue sarcoma	NA	MSS	*PMS2* del exon 8–9
	NA	Soft-tissue sarcoma	NA	MSS	*MSH2* c.942+3A>T
Kazmi et al. (2019) [25]	43, F	Malignant phyllodes tumor ^g^	MSH6 partial loss	MSS	*MSH6* ^c^
Björkman et al. (2019) [26]	31, M	Angiosarcoma	MLH1 loss	NA	*MLH1* ^c^

Note: MMR, mismatch repair; IHC, immunohistochemistry; M, male; F, female; NA, not available; NS, not specified; MSI, microsatellite instability; MSS, microsatellite stability; MSI-H, high microsatellite instability; MSI-I, indeterminate microsatellite instability (intermediate level of MSI measured by next-generation sequencing). (**a**) Sarcomas histology’s are described as in the correspondent manuscripts and may not reflect current classification of sarcomas. (**b**) The use of standard Human Genome Variation Society (HGVS) nomenclature was not possible for some variants due to the incompleteness of descriptions in the articles. (**c**) Mutation not specified. (**d**) In this study, eight sarcomas were diagnosed in five families (three with *MLH1* mutation and two with *MSH2* mutation), but no detailed information about age at disease diagnosis, tumor histology, IHC findings, or confirmation by genetic testing was provided for some cases, so they were not included. (**e**) Muir–Torre syndrome. (**f**) Inferred by clinical diagnosis of Muir–Torre and loss of *MSH2* expression. (**g**) Malignant phyllodes tumor with stromal or sarcomatous overgrowth.

**Table 3:** Compiled results of genetic testing, MMR immunohistochemistry, and MSI analysis of sarcoma cases in LS patients from the literature review.

Variable/Gene	*MSH2*	*MSH6*	*MLH1*	*PMS2*	Total
Germline mutations	25 (58.1%)	5 (11.6%)	12 (27.9%)	1 (2.3%)	43 (100%)
MMR loss and/or MSI-H/I	21 (70%)	3 (10%)	6 (20%)	0 (0%)	30 (100%)
MMR intact and/or MSS	1 (20%)	1 (20%)	2 (40%)	1 (20%)	5 (100%)
Not available	3	1	4	0	

Note: MMR, mismatch repair; MSS, microsatellite stability; MSI-H/I, high or indeterminate microsatellite instability.

## Supplementary Materials

The following are available online at https://www.mdpi.com/2072-6694/12/7/1848/s1: Table S1: VUSs found among the 14 patients with germline testing. Figure S1: *MLH1* promoter methylation analysis of patient SLS6. Figure S2: Sanger sequencing of tumor and blood DNAs from patient SLS1.
