# Supplementary material for: Clinical and Molecular Assessment of Patients with Lynch Syndrome and Sarcomas Underpinning the Association with MSH2 Germline Pathogenic Variants
Source: Cancers (Basel). 2020 Jul 9;12(7):1848. doi: 10.3390/cancers12071848 (PMC7408879; doi:10.3390/cancers12071848)
Supplement: Supplementary file 1 [file cancers-12-01848-s001.pdf]

Supplementary Materials: Clinical and Molecular Assessment of Patients with Lynch Syndrome and Sarcomas Underpinnig the Association with *MSH2* Germline Pathogenic Variants

Nathália de Angelis de Carvalho, Bianca Naomi Niitsuma, Vanessa Nascimento Kozak, Felipe D'almeida Costa, Mariana Petaccia de Macedo, Bruna Elisa Catin Kupper, Maria Letícia Gobo Silva, Maria Nirvana Formiga, Sahlua Miguel Volc, Samuel Aguiar Junior, Edenir Inez Palmero, José Cláudio Casali da Rocha, Dirce Maria Carraro and Giovana Tardin Torrezan

Table S1. VUSs found among the 14 patients with germline testing.

Patient	Gene	Variant	Classification	Rs dbSNP	GnomAD Frequency
SLS 2	*RECQL4*	c.3238G>T; p.Val1080Leu	VUS	Not available	Not available
SLS 4	*RECQL4*	c.3055+5G>A	VUS	rs377031190	T = 0.00025 (61/241496, GnomAD)
	SDHD	c.158C>T; p.Pro53Leu	VUS	rs149516118	T = 0.00004 (10/246202, GnomAD)
	CHEK2	c.1685G>T; p.Arg562Leu	VUS	rs587780180	A = 0.0001 (2/30978, GnomAD)
SLS 5	*ALK*	c.4573_4575delAAG; p.Lys1525del	VUS	rs755556501	delCTT = 0.00014 (34/251202, GnomAD)
	BRCA2	c.4930G>C; p.Glu1644Gln	VUS	rs1555283989	Not available
	FANCA	c.3430C>T; p.Arg1144Trp	VUS	rs143671872	A = 0.00052 (129/246118, GnomAD)
SLS 6	*PALB2*	c.2228A>G; p.Tyr743Cys	VUS	rs141749524	C = 0.00009 (22/246272, GnomAD)
	TP53	c.145G>C; p.Asp49His	VUS *	rs587780728	G = 0.00001 (2/246194, GnomAD)
SLS 8	*FANCD2*	c.2180C>T; p.Pro727Leu	VUS	rs146509445	T = 0.00010 (24/246266, GnomAD)
	PMS2	c.1004A>G; p.Asn335Ser	VUS	rs200513014	C = 0.00029 (70/245586, GnomAD)
	FANCM	c.374T>C; p.Met125Thr	VUS	rs780601895	C = 0.00000 (1/246232, GnomAD)
SLS 9	*CHEK2*	c.1248G>C; p.Lys416Asn	VUS *	Not available	Not available
SLS 10	*PMS2*	c.2264T>C; p.Ile755Thr	VUS	rs386833410	G = 0.00001 (3/244428, GnomAD)
	CDKN2A	c.170C>A; p.Ala57Asp	VUS	rs372266620	A = 0.00009 (20/217108, GnomAD)
	FANCB	c.1817G>A; p.Ser606Asn	VUS	rs148560784	T = 0.00006 (10/173691, GnomAD)
SLS 14	*SDHB*	c.739A>G; p.Met247Val	VUS	rs200896502	C = 0.00003 (7/246204, GnomAD)
	FANCL	c.1111_1114dupATTA, p.Thr372Asnfs	VUS	rs759217526	dupAATT = 0.00297 (727/244506, GnomAD)
SLS 15	*FANCD2*	c.3371G>C; p.Ser1124Thr	VUS	rs960135908	Not available
	APC	c.5161G>C; p.Gly1721Arg	VUS	rs1561598083	Not available
	NBN	c.511A>G; p.Ile171Val	VUS	rs61754966	C = 0.00121 (298/246104, GnomAD)
SLS 16	*PRF1*	c.755A>G; p.Asn252Ser	VUS	rs28933375	C = 0.00499 (1229/246216, GnomAD)
SLS 18	No relevant variants were detected in this patient.				
SLS 19	No VUS were detected in this patient; 2 P/LP variants were detected (manuscript table 1)				
SLS 20	No relevant variants were detected in this patient.				
SLS 21	*FANCI*	c.3473G>T; p.Cys1158Phe	VUS	rs199502679	T = 0.00011 (27/246254, GnomAD)
	SMARCB1	c.776C>T; p.Thr259Met	VUS	rs751957685	T = 0.00002 (4/246172, GnomAD)

* Variants classified as Pathogenic or Likely Pathogenic by Varsome Software.

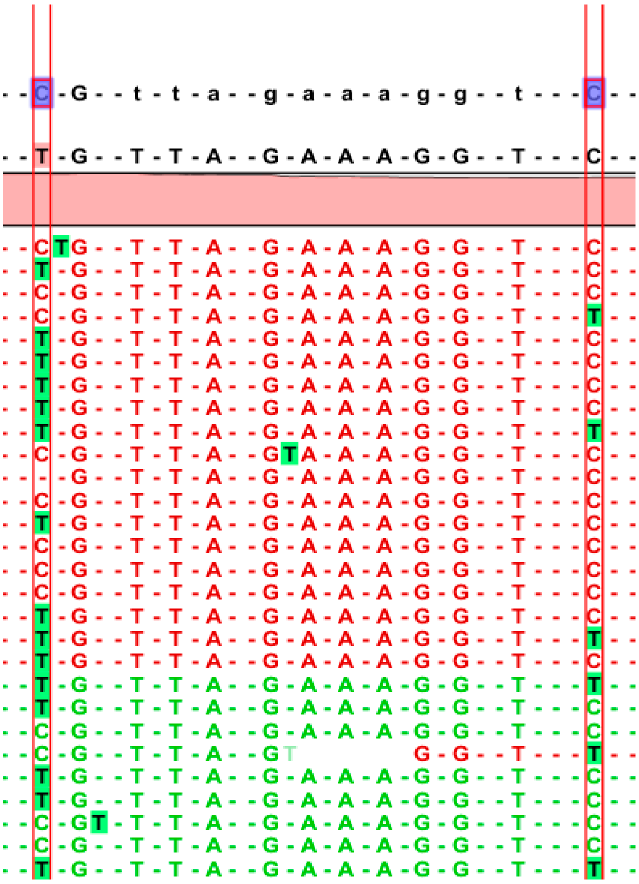

Figure S1. *MLH1* promoter methylation analysis of patient SLS6. Bisulfite-converted colorectal cancer tumor DNA from this patient was analyzed through NGS sequencing. The panel bellow shows the presence of retained C bases in CpG dinucleotides, demonstrating 41% of methylation.

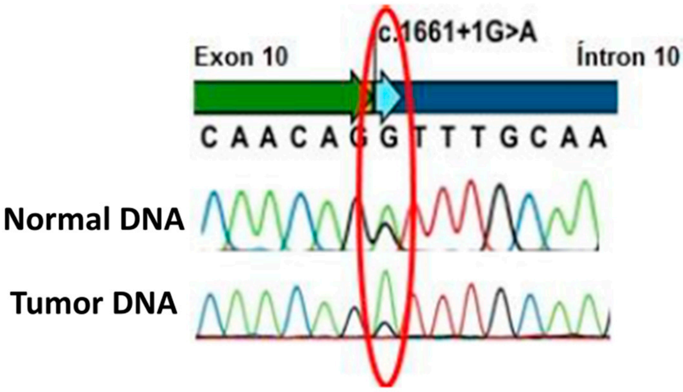

Figure S2. Sanger sequencing of tumor and blood DNAs from patient SLS1, showing the loss of heterozygosity of the *MSH2* c.1661+1G>A variant in the tumor.

© 2020 by the authors. Licensee MDPI, Basel, Switzerland. This article is an open access article distributed under the terms and conditions of the Creative Commons Attribution (CC BY) license (http://creativecommons.org/licenses/by/4.0/).
